# Influence of aridity and salinity on plant nutrients scales up from species to community level in a desert ecosystem

**DOI:** 10.1038/s41598-017-07240-6

**Published:** 2017-07-28

**Authors:** Yanming Gong, Guanghui Lv, Zhenjie Guo, Yue Chen, Jing Cao

**Affiliations:** 1 Xinjiang Key Laboratory of Oasis Ecology, Xinjiang University, Urumqi, 830046 China; 2 College of Resources and Environment Science, Xinjiang University, Urumqi, 830046 China; 3 Key Laboratory of Biogeography and Bioresources in Arid Land, Xinjiang Institute of Ecology and Geography, Chinese Academy of Sciences, Urumqi, 830011 China

## Abstract

Soil moisture and salt play key roles in regulating desert plant nutrient cycles on a local scale. However, information on the response of plant nutrient stoichiometric patterns to soil water and salt gradients is limited. Here, we assessed leaf N and P levels of 18 species of desert plants and measured the corresponding soil nutrient, water and salt concentrations, at four dry sites, five humid-saline sites and four humid-non-saline sites (reference sites) along a transect in a temperate desert in Xinjiang Province, northwest China. Our results indicated that the desert plants had lower N and P concentrations and higher N:P mass ratios in dry and humid-saline sites than in the humid-non-saline sites. Unlike the single-factor effect of salinity driving the plasticity of species N concentration, aridity and salinity interacted in their impact on the plasticity of plant P and the N:P ratio. Moreover, the plant community N and P concentrations and N:P ratio exhibited significant positive linear and nonlinear correlations with soil moisture in shallow and deep soil, respectively. Aridity reduced the N plasticity and increased P plasticity of the plant community. The results strongly supported the hypothesis that soil moisture and salt concentration were the dominant drivers of leaf N and P concentrations and their plasticity across species and community scales.

## Introduction

Nitrogen and phosphorus (hereafter, N and P) are nutrients that are essential for plant growth, metabolism, and the regulation of reproduction in terrestrial ecosystems^1, 2^. Plant N is closely associated with photosynthesis, plant productivity, and litter decomposition^3^, whereas plant P is a crucial component of genetic material, energy storage, and cellular structures^2, 4, 5^. A large body of research shows that plant N and P stoichiometry can be a focal indicator of ecosystem function, nutrient limitation, and environmental stress^2, 6^. N is important for plant growth and ecosystem processes in temperate and boreal regions^7^. Additional studies have shown that P deficiency, as well as biomass N:P ratios, reflect the balance between the uptake and loss of N and P in agricultural ecosystems^8^. N:P ratios in the organs of plants can be adjusted by internal nutrient translocation^6^. At the community level, N:P ratios can depend on plant diversity and species composition. Therefore, understanding the adaptation of plant N and P concentrations and their stoichiometry under environmental stressors has become a focus for both plant physiologists and ecologists^1, 6^.

Ecological stoichiometric research focuses on the N and P concentrations in primary producers, which may closely match environmental nutrient availability^9^. In addition, plants generally have conservative stoichiometry relative to the elemental heterogeneity of their environment^10^, such that the acquisition of N and P constrains the response of both individuals and communities to perturbation^11–13^. Nonetheless, plants are stoichiometric plastic, able to shift their elemental balance in response to environmental stress^14^. Therefore, the response of an ecosystem to environmental change, from the gene-expression level to macroscale processes, would ultimately be limited by its ability to adjust to elemental changes. Thus, shifting of stoichiometric flexibility can occur at different scales, from individual organs at the physiological level (e.g. changes in partitioning and uptake strategies) to the species level in communities and ecosystems (e.g. changes in species composition). For instance, at the level of an individual plant, stoichiometric flexibility can manifest as an adjustment in species’ organ or tissue allocation patterns^15^, resulting in differences in the response of individual species to the same environmental gradient, even among closely-related species^16^. At the community level, changes in species diversity, abundance, and trophic interactions can alter the stoichiometric composition of an ecosystem^17^.

Nutrient concentrations in plant tissues depend on inherent plant physiological traits and nutrient availability in the soil^18–20^. In most soils, N becomes available via biological N fixation and atmospheric deposition, whereas the primary source of P is rock weathering. Both nutrients are recycled via the decomposition of soil organic matter^17^. Although soil nutrients and microbiota are the main drivers of leaf nutrient concentrations^21, 22^, other soil properties and processes (for example, soil moisture and soil salt content) also determine the availability of nutrients to plants^23–25^. A recent study showed that variation in soil characteristics had more profound immediate effects on plant stoichiometry than did climate at the regional scale^26^.

As a result of the co-limitations of water content and nutrient availability in the soil, and adaptation to low-nutrient conditions, researchers have hypothesized that desert plants should show little plasticity in their N:P stoichiometry^27^ and should maintain low nutrient uptake to their tissues^28^. A previous study indicated that low soil moisture coupled with high soil alkalinity acted to decrease both soil N and P availability in desert ecosystems^29^. There was also good evidence that nutrients can sometimes limit plant growth under semi-arid conditions^30^. However, research in a saltbrush scrub community along a salinity-alkalinity gradient showed that, although N and P are expected to be growth-limiting in deserts, no relation was observed between growth and leaf N or P concentration^31^. Nevertheless, little is known about whether desert plant species can maintain flexible stoichiometries along their natural ranges of distribution in response to aridity and salinity gradients. Hence, investigating the degree to which individual desert plants, and the community, as a whole, adjust their internal stoichiometries in response to environmental changes (aridity or salinity) is necessary to further understand stoichiometric flexibility at both the individual plant and community levels, and its ecological significance.

In the current study, we tested the response of leaf N and P stoichiometry to drought (indicated by soil moisture content) and salinity (indicated by soil soluble salt content), for 18 plant species at thirteen sites in the Ebinur Lake Wetland Nature Reserve in the Xinjiang Uygur Autonomous Region, China. Our aim was to understand the effects of potential drivers (soil water and soluble salt content, and soil N and P levels in shallow and deep soil layers) on leaf N and P and the N:P stoichiometry of individual plant species and the community as a whole across the study sites. We addressed three main questions: (1) how does leaf stoichiometry in individual plants, and within the community as a whole, change along the transect from arid to saline conditions? (2) how are soil moisture, soil salt content, and soil fertility associated with these patterns? (3) how does variation in the community stoichiometry reflect different responses to dry and saline environments?

## Material and Methods

### Study area and sampling sites

**Figure 1:**
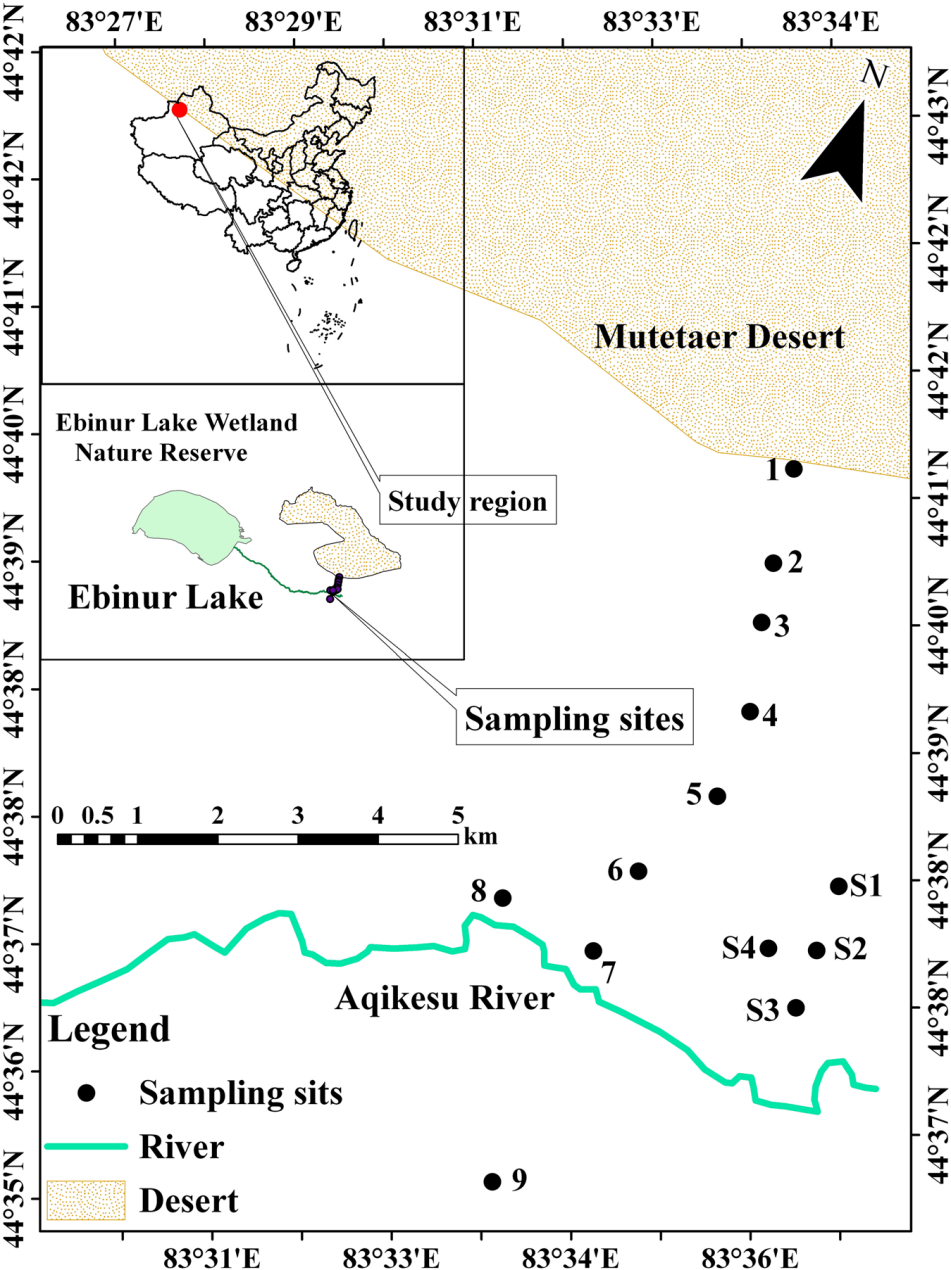
Sampling sites. An 8-km long transect was sampled in the Ebinur Lake Wetland Nature Reserve in Xinjiang Uygur Autonomous Region of China. A total of 13 sampling sites were selected along this gradient. Dry sampling sites were nos 1–3, humid-saline sites were nos 4–9, humid-non-saline sites were nos S1–S4. This figure was originally generated using the software ArcGIS 10.0 (http://www.esri.com/software/arcgis/arcgisonline).

In early July 2015, we established an approximately 8-km long north–south transect in the Ebinur Lake Wetland Nature Reserve in Xinjiang Uygur Autonomous Region, China (44°30′–45°09′N, 82°36′–83°50′E) (Fig. 1). The study area is characterized by a gentle topography, with elevations ranging from 290 to 331 m above sea level. The region has a homogeneous continental climate, characterized by extremely dry conditions and sparse rainfall. The mean annual air temperature ranges from 6.6 to 7.8 °C, the mean annual precipitation is less than 100 mm, and the potential evaporation is more than 1600 mm (Fig. S1). Thus, no difference in rainfall was observed along the transect, enabling us to sample across two extreme soil environments: extreme drought on the verge of the Mutter Desert, and high soil salinity around the Aqikesu River. Soil type is predominantly desert soil belonging to the Kastanozem soil group in the Food and Agriculture Organization classification system^32^. The sampling sites selected were deemed to be representative of natural conditions without any grazing activity or exposure to other anthropogenic disturbances. Thirteen sampling sites were investigated along the transect with about a 1-km gap between each sampling site (Fig. 1, sites 1–9, S1–S4). Sampling locations were GPS-referenced with latitude, longitude, and elevation (eTrex Venture, Garmin, Olathe, KS, USA).

### Sampling and measurements

At each of the thirteen sites, ten 10 m × 10 m main plots were selected in a direction perpendicular to the transect, and each plot was separated from the next by an interval of 10 m. Collection and measurement of samples were conducted during early July 2015 (with the exception of sites S1–S4, where sampling took place in July 2014). The mean plant height of each plant species in each plot was measured with a ruler. Fresh and mature foliar samples (hereafter referred to as ‘foliar’, Table 1) were collected from five to ten individual plants of each species in each site and then stored in separate paper bags (each bag contained the tissue from one plant of the species). For each species, leaves that were similar in terms of their size, shape, and color were sampled. To reduce the influences of dust or soil, foliar samples were rinsed with deionized water at least twice. Plant materials in the field were dried at 105 °C for 30 min in a portable drying oven to minimize respiration and decomposition losses, and were later completely oven dried at 70 °C to a constant weight in the laboratory. In total, 357 plant samples were collected, belonging to 18 plant species (Table 1) across the 13 study sites. After removing the litter layer, soil samples were randomly collected at two soil depths (shallow soil layer [0–20 cm] and deep soil layer [20–100 cm]), with three replicates at each sampling site. The three samples from each soil layer were mixed evenly. Subsamples of each soil sample were stored at 4 °C immediately after collection to determine the initial gravimetric moisture content. This was measured by drying the weighed samples at 105 °C for 48 h to a constant weight.

**Table 1:** Plant species and soil factors at thirteen sites. Values were means and error variances (in parentheses). The values for soil factors were means and standard errors at two soil depths in nine sites. Common superscript letters on the mean values within a column indicate no significant difference at P > 0.05 using LSD tests.

Site	Spieces (Abbreviation)	Soil water content (%)		Soil soluble salt content (g kg^−1^)		Soil total nitrogen concentration (mg g^−1^)		Soil total phosphorus concentration (mg g^−1^)		Soil organic carbon content (g kg^−1^)	
		0–20 cm	20–100 cm	0–20 cm	20–100 cm	0–20 cm	20–100 cm	0–20 cm	20–100 cm	0–20 cm	20–100 cm
1	*Ha*, *Ce*	1.63^a^ (0.019)	1.28^a^ (0.015)	0.95^a^ (0.144)	2.76^a^ (0.352)	0.25^ab^ (0.024)	0.22^ab^ (0.020)	0.33^b^ (0.043)	0.28^bc^ (0.015)	1.41^a^ (0.016)	1.29^a^ (0.007)
2	*Ha*, *Ce*, *Ph*, *Ss*	1.45^a^ (0.132)	2.63^b^ (0.021)	3.15^ab^ (0.360)	4.27^a^ (0.207)	0.25^ab^ (0.011)	0.24^abc^ (0.006)	0.32^b^ (0.014)	0.26^ab^ (0.014)	1.39^a^ (0.079)	1.37^a^ (0.028)
3	*Ha*, *Ce*, *Ph*, *Ss*, *As*	1.21^a^ (0.034)	1.69^a^ (0.062)	1.23^a^ (0.105)	3.03^a^ (0.062)	0.26^ab^ (0.007)	0.26^bc^ (0.028)	0.27^a^ (0.014)	0.26^ab^ (0.006)	1.73^ab^ (0.036)	1.38^a^ (0.033)
4	*Ha*, *Ce*, *Ph*, *Ss*, *As*, *Rs*, *Ns*, *Pa*, *Pe*	0.90^a^ (0.008)	3.65^c^ (0.058)	7.79^ab^ (0.264)	7.30^b^ (0.564)	0.20^a^ (0.009)	0.18^a^ (0.002)	0.28^a^ (0.011)	0.27^bc^ (0.012)	1.77^ab^ (0.111)	1.61^a^ (0.036)
5	*Ph*, *As*, *Rs*, *Ns*, *Pa*, *Pe*, *Tr*, *Kc*, *Kf*	1.20^a^ (0.176)	3.95^c^ (0.247)	15.73^b^ (0.645)	9.18^b^ (0.194)	0.22^a^ (0.010)	0.19^a^ (0.004)	0.28^a^ (0.023)	0.24^ab^ (0.015)	1.87^ab^ (0.074)	1.46^a^ (0.074)
6	*Ha*, *Ph*, *As*, *Rs*, *Ns*, *Pa*, *Pe*, *Kf*, *Hs*	2.96^a^ (0.226)	9.04^d^ (0.266)	33.01^c^ (2.331)	19.73^c^ (0.414)	0.26^ab^ (0.007)	0.24^abc^ (0.015)	0.32^b^ (0.024)	0.31^c^ (0.015)	3.01^abc^ (0.132)	2.19^b^ (0.064)
7	*Ha*, *Ph*, *Pa*,*Pe*, *Hh*	5.29^b^ (0.162)	10.40^e^ (0.119)	41.41^c^ (1.091)	18.21^c^ (0.660)	0.27^ab^ (0.010)	0.31 ^cd^ (0.019)	0.29^ab^ (0.003)	0.28^bc^ (0.004)	3.65^bc^ (0.186)	2.49^b^ (0.116)
8	*Ns*, *Pe*, *Av*, *Sm*, *Gu*	20.03^e^ (1.314)	21.13^h^ (0.522)	138.63^e^ (8.207)	19.51^c^ (0.414)	2.01^c^ (0.064)	0.99^e^ (0.035)	0.32^b^ (0.007)	0.22^a^ (0.012)	29.43^e^ (1.871)	4.98^d^ (0.234)
9	*Pa*, *Tr*, *Kc*, *Hh*, *Hc*, *Hs*	9.79^c^ (1.396)	18.55^g^ (0.122)	88.68^d^ (8.742)	32.94^d^ (2.240)	0.32^b^ (0.025)	0.36^d^ (0.030)	0.28^a^ (0.006)	0.25^ab^ (0.003)	4.50^c^ (0.228)	3.66^c^ (0.159)
S1	*Ha*, *Ns*, *Pa*, *Pe*, *Hh*, *Av*, *Sm*	14.09^d^ (1.156)	13.62^f^ (0.985)	6.32^ab^ (0.237)	4.08^a^ (0.316)	0.27^ab^ (0.005)	0.23^ab^ (0.015)	0.27^a^ (0.009)	0.23^a^ (0.006)	5.83^c^ (0.368)	2.15^b^ (0.113)
S2	*Ha*, *Ns*, *Pa*, *Tr*, *Kf*, *Av*	10.35^c^ (1.205)	10.19^e^ (0.877)	4.61^ab^ (0.341)	4.26^a^ (1.301)	0.21^a^ (0.011)	0.20^a^ (0.006)	0.29^ab^ (0.017)	0.29^bc^ (0.014)	1.45^a^ (0.096)	1.05^a^ (0.008)
S3	*Ha*, *As*, *Ns*, *Pe*, *Tr*, *Kc*, *Av*, *Hs*	9.29^c^ (0.505)	10.91^e^ (0.743)	3.47^ab^ (0.054)	4.04^a^ (0.124)	0.21^a^ (0.009)	0.28^bc^ (0.018)	0.26^a^ (0.005)	0.24^ab^ (0.011)	2.65^ab^ (0.052)	1.85^ab^ (0.006)
S4	*Ha*, *As*, *Ns*, *Pe*, *Kf*, *Av*, *Sm*, *Gu*	14.38^d^ (1.782)	13.35^f^ (1.338)	5.10^ab^ (0.423)	4.56^a^ (0.183)	0.24^ab^ (0.010)	0.22^ab^ (0.012)	0.27^a^ (0.019)	0.25^ab^ (0.008)	7.11^d^ (0.167)	3.08^bc^ (0.021)

Note: Ha*, Haloxylon ammodendron. Ce*, Calligonum ebinuricum. Ph, Poacynum hendersonii. Ss, Seriphidium santolinum. As, Alhagi sparsifolia. Rs*, Reaumuria soongorica. Ns, Nitraria sibirica. Pa, Phragmites australis. Pe, Populus euphratica. Tr*, Tamarix ramosissima. Kc, Karelinia caspica. Kf*, Kalidium foliatum. Hh, Halimodendron halodendron. Av, Apocynum venetum. Sm*, Suaeda microphylla. Gu, Glycyrrhiza uralensis. Hc*, Halostachys caspica. Hs*, Halocnemum strobilaceum. The asterisk (*) means that plant photosynthetic organ is assimilating shoot. Sites 1–4 are the dry s sites, sites 5–9 are the humid-saline sites, S1–S4 are the humid-non-saline sites (reference sites).

Dried plant and soil materials were ground to pass through a 1-mm sieve (Retsch MM 400; Retsch, Haan, Germany). Leaf N concentration was analyzed with a PE-2400 CHN analyzer (Perkin-Elmer, Foster City, CA, USA). Leaf P concentration was measured colorimetrically after H_2_SO_4_-H_2_O_2_-HF digestion using the molybdate/stannous chloride method^32^. The available N:P ratios were calculated from these variables at a sample level.

Soil total N concentration was analyzed with a Kjeltec System 2300 Analyzer Unit (Tecator, Höganäs, Sweden). Soil total P concentration was determined with the molybdate/ascorbic acid blue method after digestion with HClO_4_ and H_2_SO_4_ acid^33^. Soil soluble salt concentration was obtained using the weight method, in which the soil extraction liquid was dried, followed by removal of the organic matter from the dry residue using H_2_O_2_, and the resulting liquid dried again at 105–110 °C and then weighed.

### Data analysis

Before numerical and statistical analyses, all variables for each species were averaged at the plot level and all variables relating to the soil samples were averaged at the site level (Table 1). Data were tested for normality using the Kolmogorov-Smirnov test and for equality of error variance using Levene’s test. Weighted community N and P concentration and N:P ratios were calculated according to Equation 1:

1 $${y}_{j}=\sum _{i=1}^{n}({x}_{i}\times {a}_{i}\times {h}_{i})$$

where y_j_ was the weighted mean of community N or P concentration (g kg^−1^ dry mass) or the N:P ratio of the *j* site, *x*_i_ was the mean of foliar N or P concentration (g kg^−1^ dry mass) or the N:P ratio of the *i* species in the *j* site, *a*_*i*_ was the relative abundance of the *i* species in the *j* site, and *h*_*i*_ was the relative height of the *i* species in the *j* site. Linear or nonlinear regression was used to analyze the relationship between N:P ratio and both plant N and P concentration for each species in response to the soil water and salt gradients, and to examine the response of plant nutrient concentrations to soil factors (i.e. soil water, soil total salt and soil nutrient content). Through principal component analysis (PCA) of the environmental data, sites 1, 2, 3 and 4, sites 5, 6, 7, 8, and 9, and sites S1, S2, S3 and S4 were defined to be dry sites (low soil salt content, see Table 1), humid-saline sites and humid-non-saline sites, respectively (Fig. S2). One-way analysis of variance (ANOVA) was used to examine the differences in leaf N, P (content or concentration) and N:P ratios among all the plants from the dry sites, humid-saline sites and humid-non-saline sites, and differences in soil factors among all the sites. The 3-D mesh plot was also used to show the response trend in coefficient of variance (CV) of the leaf N, P and N:P ratio for each species due to soil water and salt content. We further analyzed the linear or nonlinear relationships based on the regression analysis between each of the community nutrient metrics (N, P and N:P ratio) and both soil water content and salt content. The above statistical analyses were conducted using the statistical package SPSS (PASW statistics 21.0; IBM Corporation, Armonk, NY, USA) and SigmaPlot 12.5 (SyStat Software Inc., San Jose, CA, USA). Variation in community N, P and N:P ratio was partitioned between two explanatory variable groups (soil water content [0–20 cm, 20–100 cm] and soil salt content [0–20 cm, 20–100 cm]) using a partial regression analysis with a redundancy analysis (RDA)^34^. PCA and RDA were conducted using CANOCO 5.0 (Microcomputer Power, Ithaca, NY, USA).

## Results

### Soil properties

There was great variation along the transect in the soil water content, from 0.90% to 20.03% at a soil depth of 0–20 cm and from 1.28 to 21.13% at a soil depth of 20–100 cm (Table 1). In addition, several study sites were found to be highly saline, with soil salinity contents of 138.62 and 32.94 g kg^−1^ at soil depths of 0–20 (site 8) and 20–100 cm (site 9), respectively (Table 1). Our study revealed that soil water contents in dry sites were significantly lower than those in the humid-saline sites and the humid-non-saline sites (P < 0.05), while soil salt contents of humid-saline sites were significantly higher than those of the dry sites and the humid-non-saline sites (P < 0.05, Fig. S3). The ranges of total N concentration values in the soil were 0.20–2.01 mg g^−1^ (soil depth 0–20 cm) and 0.18–0.99 mg g^−1^ (soil depth 20–100 cm), while those for total P concentration were 0.26–0.33 mg g^−1^ (soil depth 0–20 cm) and 0.22–0.29 mg g^−1^ (soil depth 20–100 cm) (Table 1). There was no significant difference in soil total nitrogen, soil total phosphorus and soil organic carbon among dry sites, humid-saline sites and humid-non-saline sites (Fig. S3).

### Changes in nutrient levels in individual plants along the soil water and salt gradients

**Figure 2:**
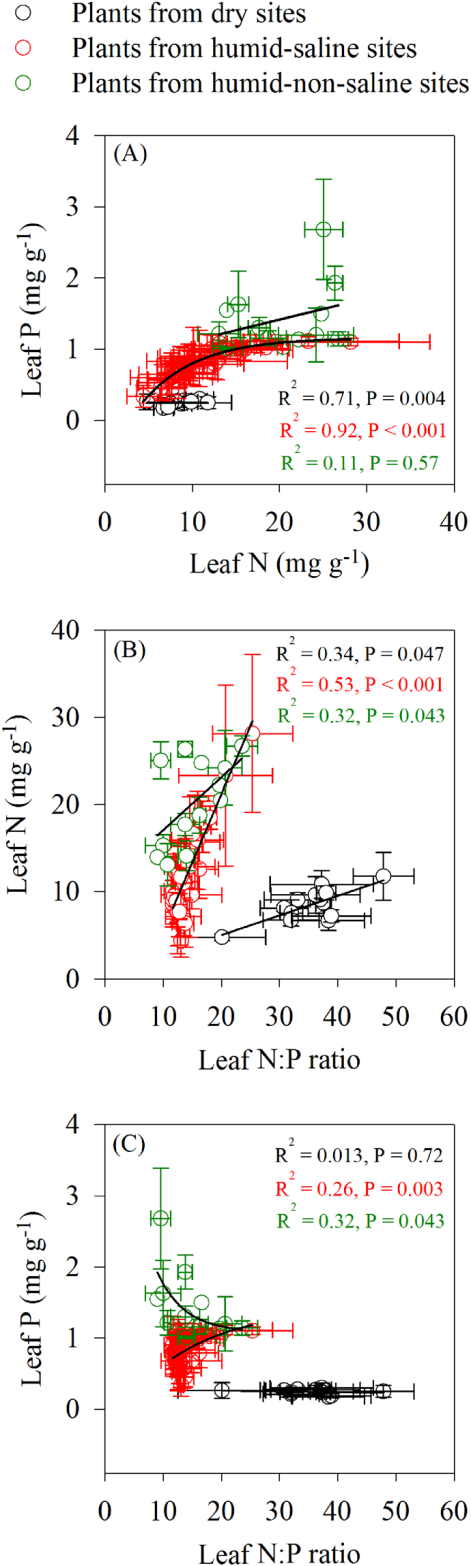
Relationships between leaf N or P concentrations and N:P ratios, respectively. The black, red and green circles represent the mean values of plant species’ N, P concentrations and N:P ratios from dry, humid-saline and humid-non-saline sites, respectively (error bars denote SE).

In the current study, nutrient levels in the leaves of eighteen plant species ranged from 4.38 mg g^−1^ to 28.25 mg g^−1^, 0.17 to 3.18 mg g^−1^, and 6.54 to 47.88 mg g^−1^ for N concentration, P concentration and N:P ratio, respectively (Fig. 2).

In dry sites and humid-saline sites, there were significant positive correlations between the species N and P concentrations, species N and N:P ratios, respectively. In the humid-saline sites and humid-non-saline sites, significant positive correlations were found between species P and N:P ratios. In contrast, there were significant negative correlations between the species P and N:P ratios in the dry sites and humid-non-saline sites, respectively (Fig. 2).

**Figure 3:**
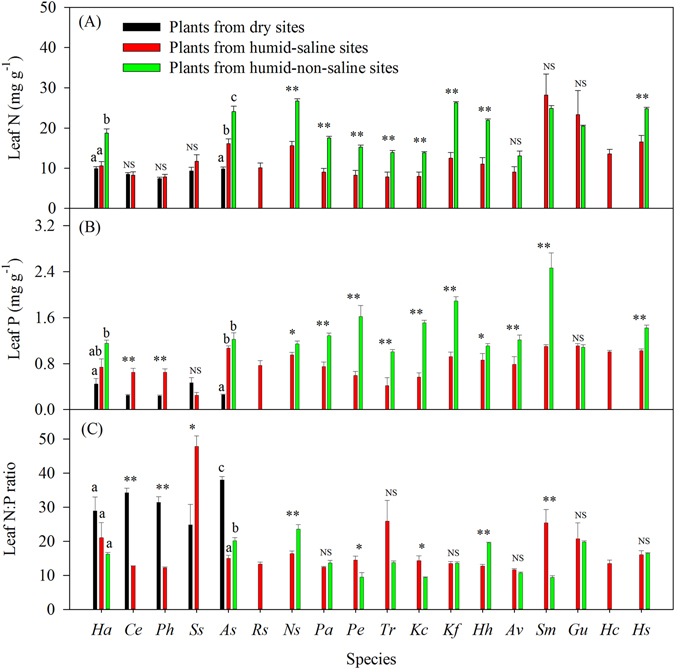
Comparisons of species stoichiometry between dry and humid-saline sites and humid-non-saline sites, respectively. Significant differences are reported from ANOVA as NS, P ≥ 0.05; *P < 0.05; **P < 0.01. Following ANOVA, multiple comparisons were conducted using Protected Least Significant Difference (LSD); any two samples with a common letter (a, b or c) are not significantly different (P > 0.05) The abbreviations of species refer to the note in Table 1.

At the species level, plant N and P concentrations were significantly lower than in the reference values (species from humid-non-saline sites) than in the leaves of Ha and As, and the plant N:P ratio was significantly higher than the reference values in the leaves of As from dry sites (Fig. 3A,B, P < 0.05). Leaf N concentrations of Ha, As, Ns, Pa, Pe, Tr, Kc, Kf, Hh and Hs from humid-saline sites were significantly lower than the reference values (P < 0.05), while leaf P concentrations of As, Ns, Pa, Pe, Tr, Kc, Kf, Hh, Av, Sm and Hs from humid-saline sites were also significantly lower than those from humid-non-saline sites (Fig. 3A,B, P < 0.05). Furthermore, plant N:P ratios from As, Ns and Hh from humid-saline sites were significantly lower than the reference values, whereas plant N:P ratios of Pe, Kc and Sm were significantly higher (Fig. 3C, P < 0.05). By analyzing the relationships between N and P concentrations and N:P mass ratios from all species on the dry, humid-saline and humid-non-saline sites, we found that the leaf N concentration from plants on the dry sites was significantly lower than that from humid-saline and humid-non saline sites (P < 0.05). Conversely, the leaf N:P ratio from the dry sites was significantly higher than that from the other sites (P < 0.05). There were also significant differences in the leaf P concentration values between the dry, humid-saline and humid-non-saline sites (P < 0.05) (Fig. S4).

**Figure 4:**
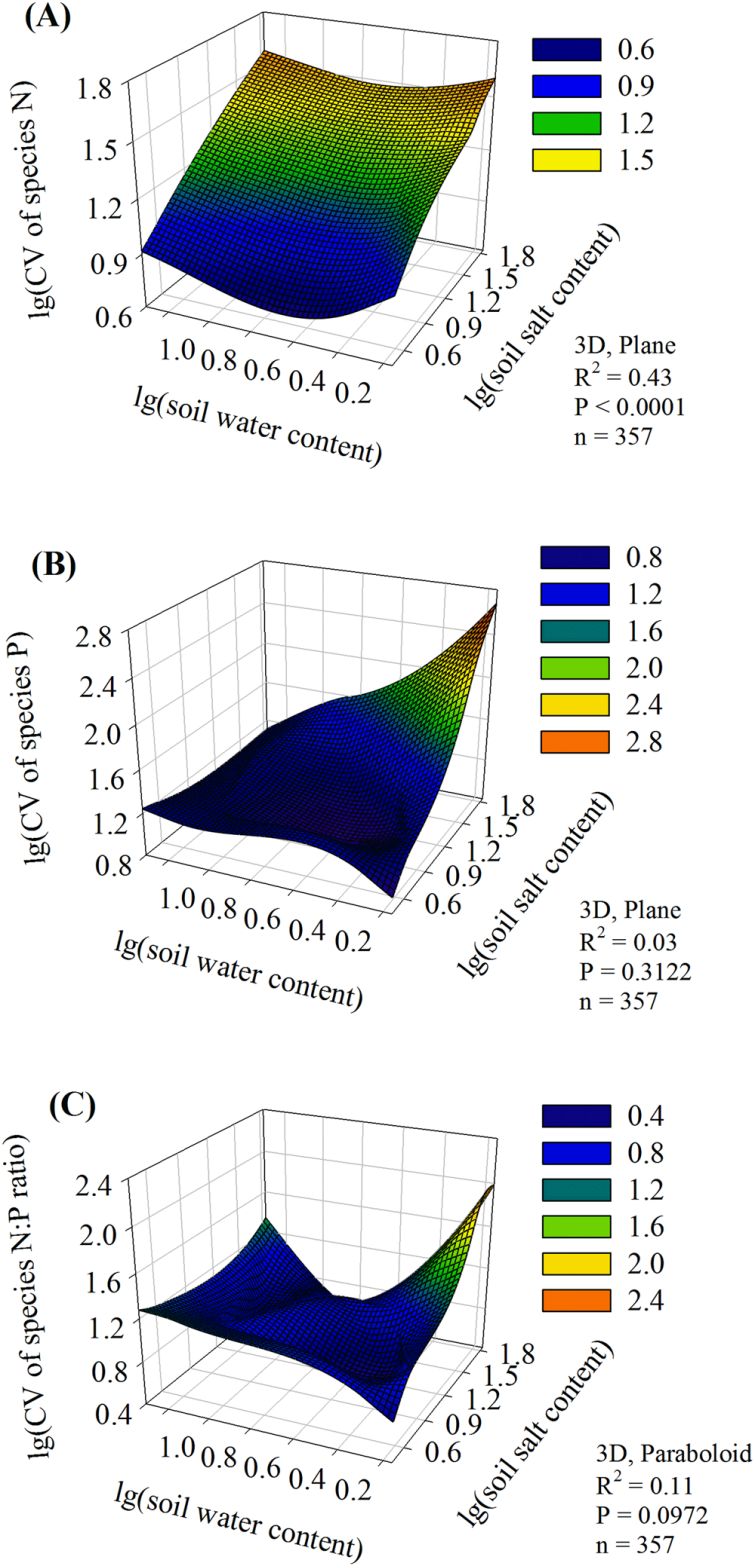
Relationships between coefficient of variance (CV) of species stoichiometry and soil water and salt contents.

Environmental variation was a strong driver of CV of N and P stoichiometry. Significant nonlinear polynomial regressions were found between CV of species N and both soil water and salt contents (R^2^ = 0.43, P < 0.05, n = 357; Fig. 4A). The nonlinear relationships included plane and paraboloid trends between CV of species’ P concentration, N:P ratio and either soil water or salt contents (Fig. 4B,C)

### Plant community nutrients in relation to edaphic factors

**Figure 5:**
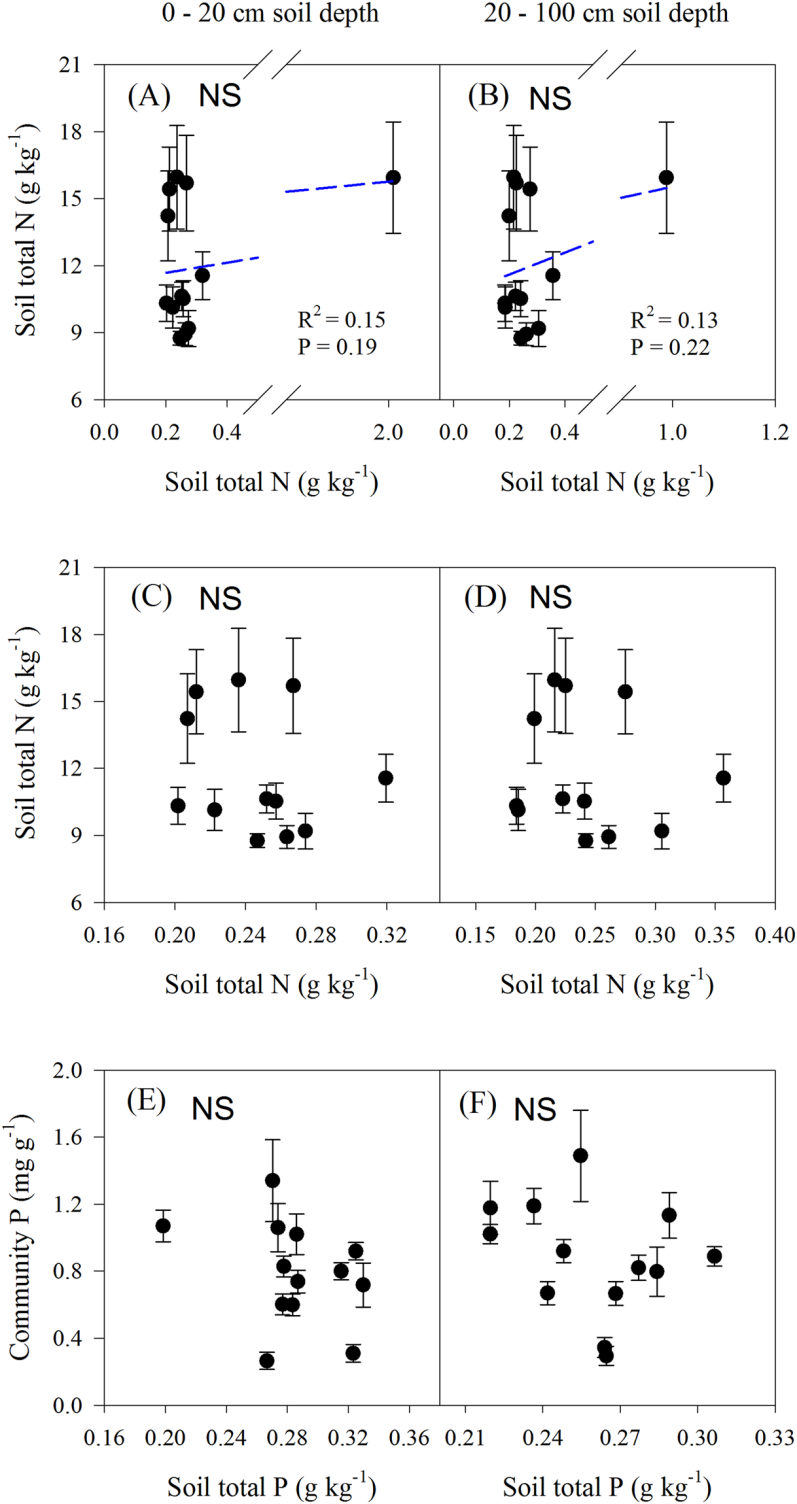
Relationships between plant community nutrients and soil nutrients across the transect. Blue solid lines represent the fitted linear regressions. Figures C and D excluded the data on soil total N at site 8 (the data were higher than others and influenced the result of the linear regression analysis).

**Figure 6:**
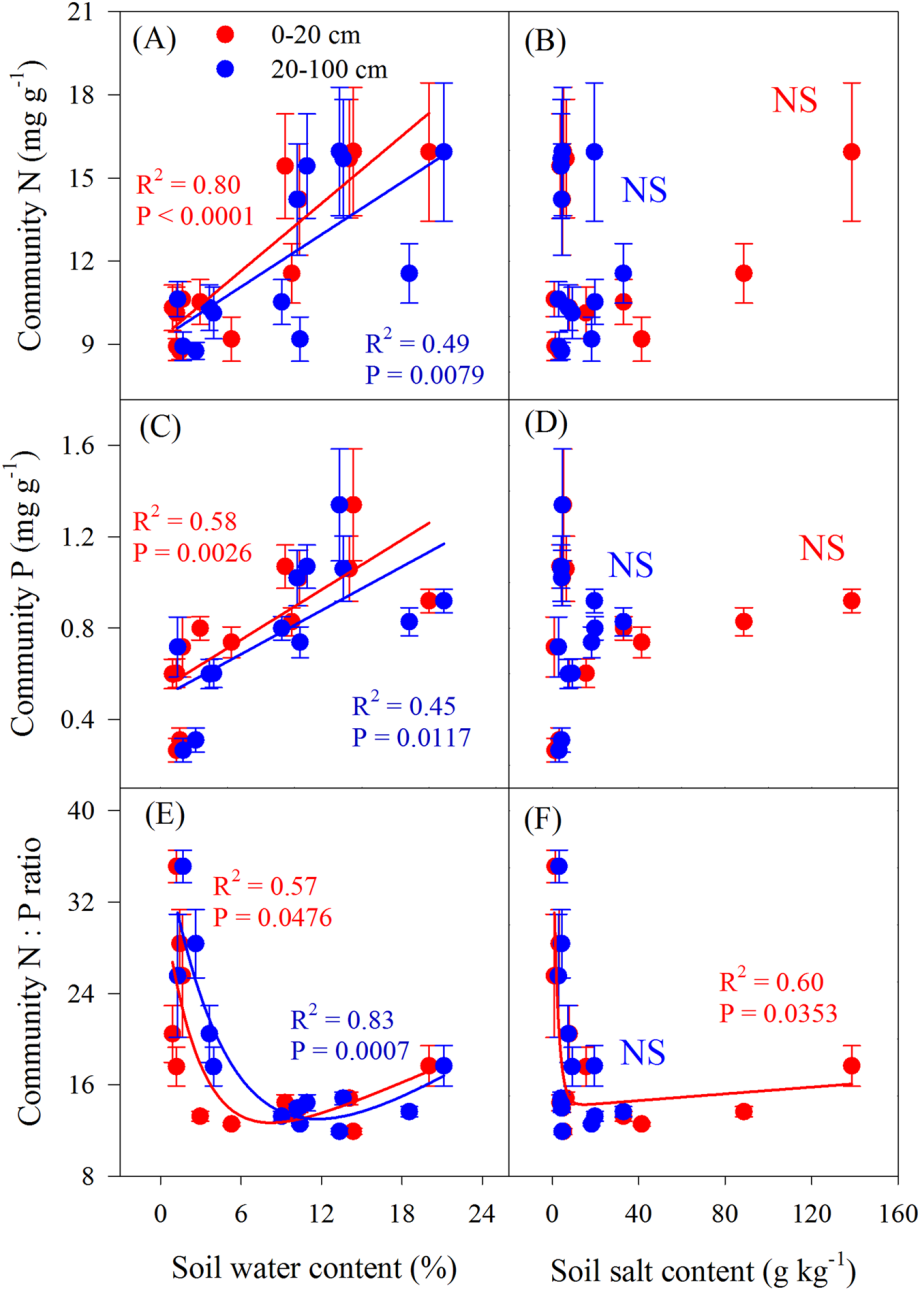
Relationships between plant community nutrients and either soil water or salt concentration.

There were no statistically significant relationships between soil total N, P or salt concentration and plant community N or P concentration at either soil depth across the transect, respectively (P > 0.05; Fig. 5, Fig. 6B,D). By contrast, significantly positive linear relationships were found between community N or P concentrations and soil water content across the transect (P < 0.05; Fig. 6A,C); significant nonlinear relationships were found between community N:P ratios and both soil water (0–20 cm and 20–100 cm, respectively) and salt content (0–20 cm) (P < 0.05; Fig. 6E,F).

**Figure 7:**
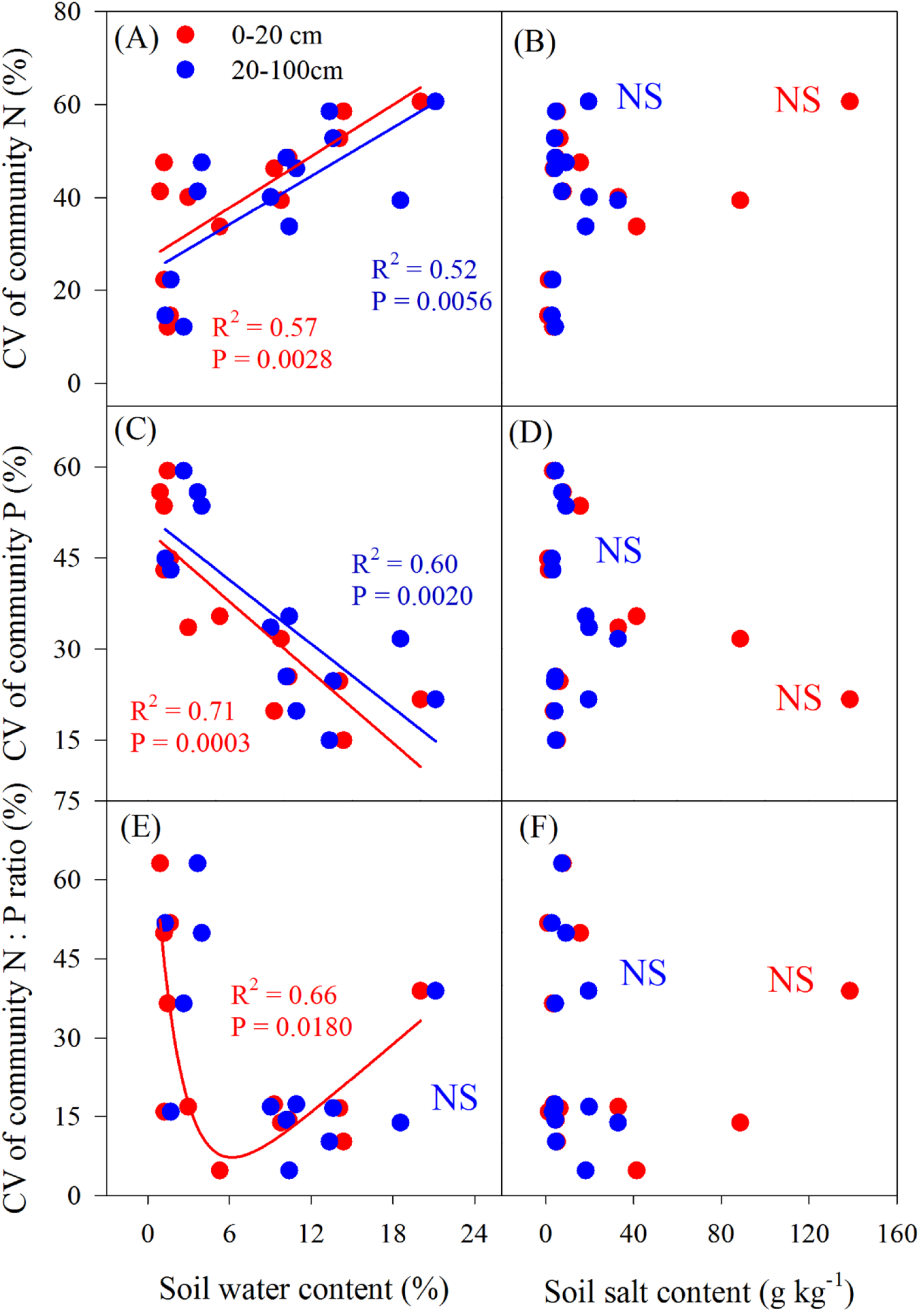
Relationships between the coefficient of variance (CV) of community nutrients and soil water and salt concentration.

There was a significant linear positive correlation between the CV of community N concentration and soil moisture (0–20, 20–100 cm) (P < 0.05; Fig. 7A), and a significant linear negative correlation between the CV of community P concentration and soil moisture (0–20 cm, 20–100 cm) (P < 0.05, Fig. 7C). In addition, a significant nonlinear regression was found between the CV of community N:P ratio and soil moisture (0–20 cm) (P < 0.05, Fig. 7E). However, there was no correlation between CV of community N, P or N:P ratio and soil salinity (Fig. 7B,D,F).

**Figure 8:**
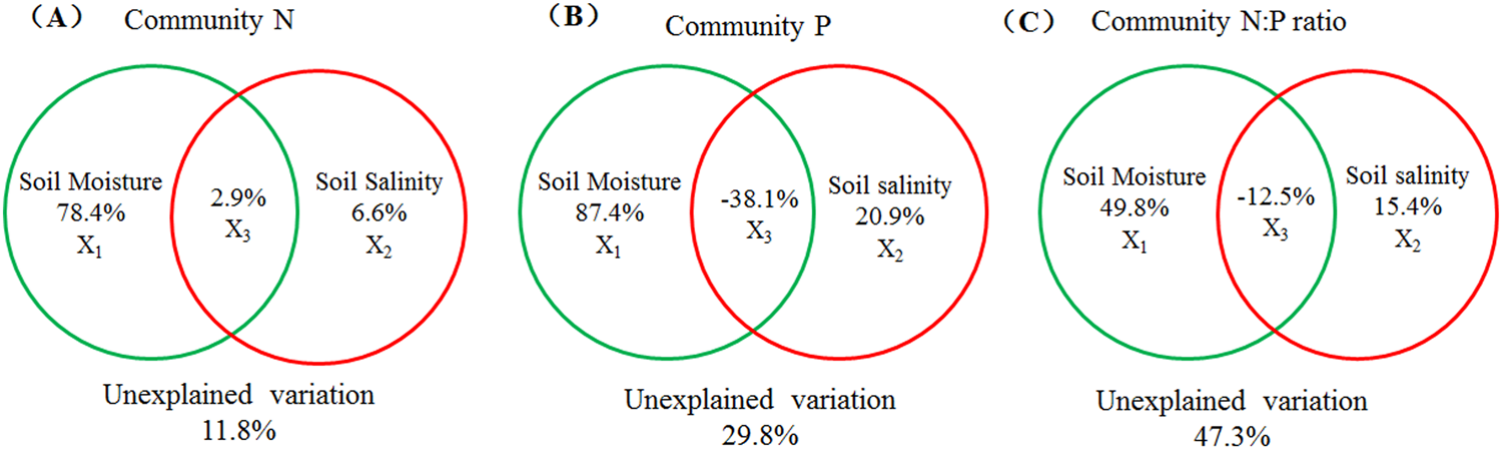
The results of variation-partitioning analysis for species and community N, P and N:P ratio. Variation-partitioning analysis led to the following four fractions: single effect of soil moisture (X1), single effect of soil salinity (X2), joint effects of soil moisture and salinity (X3), and unexplained variation.

A variation-partitioning analysis further demonstrated that community stoichiometry was largely explained by soil water content (X1, Fig. 8), while the single effect of soil salt concentration itself (X2) was small. The proportions of the variation associated with soil moisture and salt concentration were 78.4% and 6.6% for community N, respectively, 87.4% and 20.9% for community P, respectively, and 49.8% and 15.4% for community N:P ratio, respectively (Fig. 8).

## Discussion

### Relation between individual plant nutrients and soil water and salt contents

Numerous studies have reported plant stoichiometric flexibility in response to variation in soil environmental factors, and the flexibility varied with study scales^1, 6, 14, 30, 35^. Our study showed that variation in soil water and salt concentration had more profound effects than soil total N and P concentrations on plant stoichiometry at the local scale. In desert ecosystems, infrequent and low precipitation limits soil weathering, organic matter production, and mineralization^36^, leading to slow P release from primary material, low soil organic matter content, and N bound to organic matter^27^. In addition, soil salinity was aggravated by high average annual evaporation on both sides of the Aqikesu River. Furthermore, soil physical and chemical properties and soil microbiota determine the nutrient availability of individual plants^23–25^. Plant N fixation rates in arid regions have long been considered to be low because of low soil moisture and high temperatures^37^. Our study indicated that low soil moisture, coupled with high soil salinity, acted to decrease both plant leaf N and P concentrations at the species level. This is indirect evidence that soil drought and salinity inhibits the growth of individual plants, but is not consistent with many previous studies on desert ecosystems^35, 38, 39^, which showed that such plants were characterized by high N levels. In the current study, the mean leaf N concentration (11.98 mg  mg g^−1^) of the 18 plant species was lower than the range (20.09–26.46 mg g^−1^) reported for plants in terrestrial ecosystems^1, 40–42^. The mean leaf P concentration (0.73 mg g^−1^) in this study was also clearly lower than the results (1.46–1.99 mg g^−1^) reported for Chinese terrestrial and global ecosystems^1, 40–42^. On the other hand, the mean leaf N:P ratio of this study (21.32) was higher than that for plants in terrestrial ecosystems^1, 40–42^.

The plasticity of plant N concentration was associated with high-salt environments. In contrast, we did not find an obvious relationship between the plasticity of N concentration and soil moisture, which may be due to salinity-based control of N uptake (Fig. 4A). Drought and salinity interacted to influence the plasticity of species P concentration and the N:P ratio (Fig. 4B,C). Therefore, it is unlikely that the plasticity of plant N and P stoichiometry adapted to environmental variation at the species level. The current study showed that plant nutrient stoichiometry was dependent on stressors such as aridity and salinity, which might be because the gradients of soil water and salt concentrations have important roles in controlling and regulating N:P stoichiometry in dry or saline environments^43^.

### Aridity and salinity, rather than soil fertility, control plant community nutrient stoichiometry

Soil environmental changes affected leaf nutrient stoichiometry at the local scale^39^. In addition, there was variability in the leaf N and P concentrations and in the N:P ratio as a result of the different levels of adaptability of individual plants and the community as a whole to dry or saline conditions. Research showed that leaf nutrient stoichiometry was at least partially controlled by total soil P levels in the Alxa desert^35^. In the current study, the environmental extremes also reduced the importance of soil nutrients in determining N and P concentrations and N:P ratios from the species to the community levels.

The results of the current study showed that drought, especially in the upper soil horizons (0–20 cm), inhibited the uptake of nutrients by plants of the desert community, affecting N:P stoichiometry. There was no significant correlation between plant community nutrient stoichiometry and soil salinity in the desert ecosystem. Variation-partitioning analysis also revealed that soil moisture had important effects on community nutrient stoichiometry, but relatively few single-factor effects were observed for soil salinity (Fig. 8). Unraveling the effects of aridity and salinity by variation-partitioning analysis, we also observed that soil salt stress reduced the impact of soil moisture on community P concentrations and N:P ratios, with [salt x moisture] interactions having negative effects on leaf P (Fig. 8). A previous study showed that soil-available nutrients, but not salinity, were potential drivers of the leaf N:P stoichiometry in an arid-saline environment^38^, which is not consistent with our conclusions.

Our results indicated that drought reduced change in the plasticity of community N stoichiometry, and increased the plasticity of community P along this transect. Furthermore, the impact of drought on the plasticity of community N was greater in the shallow soil depths (0–20 cm); on the contrary, the impact on the plasticity of community P tend to be due to moisture in deep soil horizons (20–100 cm). However, we did not find a statistically significant effect of soil salinity on community N and P stoichiometry, which may be due to low variability in the stoichiometry.

Drought and salt stresses are known to be instrumental in shaping community and species distributions along abiotic stress gradients^31^. Also, community turnover constrained the effect of diversity on plant stoichiometry across the transect^6, 17^. Plant N and P uptake were affected by soil moisture and salt concentrations, which could have resulted in total soil N and P being poor indicators of soil nutrient availability^35^. Species coexistence in the current study appears to be facilitated by their co-adaptability to drought and salinity, with some species able to adapt to either the dry or the saline environment, while others can adapt to both. Therefore, further work will be required to determine the physiological mechanisms operating to adapt to drought and salinity in the different species, and to establish the physiological basis for the observed plant nutrient stoichiometric patterns.

## Conclusions

In the current study, we found that soil water and salt contents interacted to regulate plant ecological nutrient stoichiometry from the species to the community level in the 0–1 m soil profile in the desert ecosystem studied here. By reducing leaf N and P concentrations and increasing the plasticity of these variables, plants have adapted to dry and saline environments. In addition, soil moisture played a more important role than soil salinity in regulating desert plant nutrient levels^44^.

## Electronic supplementary material

Supplementary Information
