# Supplementary material for: Influence of aridity and salinity on plant nutrients scales up from species to community level in a desert ecosystem
Source: Sci Rep. 2017 Jul 28;7:6811. doi: 10.1038/s41598-017-07240-6 (PMC5533738; doi:10.1038/s41598-017-07240-6)
Supplement: Supplementary file 1 — Supplementary Information [file 41598_2017_7240_MOESM1_ESM.docx]

Supplementary Materials

**Influence of aridity and salinity on plant nutrients scales up from species to community level in a desert ecosystem**

Yanming Gong^1,2,3,+^, Guanghui Lv^1,2,*^, Zhenjie Guo^1^^,2,+^, Yue Chen^1,2^, Jing Cao^1,2^

^1^ Xinjiang Key Laboratory of Oasis Ecology, Xinjiang University, Urumqi, 830046, China.

^2^ College of Resources and Environment Science, Xinjiang University, Urumqi, 830046, China.

^3^ Key Laboratory of Biogeography and Bioresources in Arid Land, Xinjiang Institute of Ecology and Geography, Chinese Academy of Sciences, Urumqi, 830011, China.

^*^Correspondence and requests for materials should be addressed to L.G.H. (email: ecology2007@163.com)

^+^These authors contributed equally to this work.





**Figure S1** Precipitation and air temperature from 2014 to 2016 in the study local.


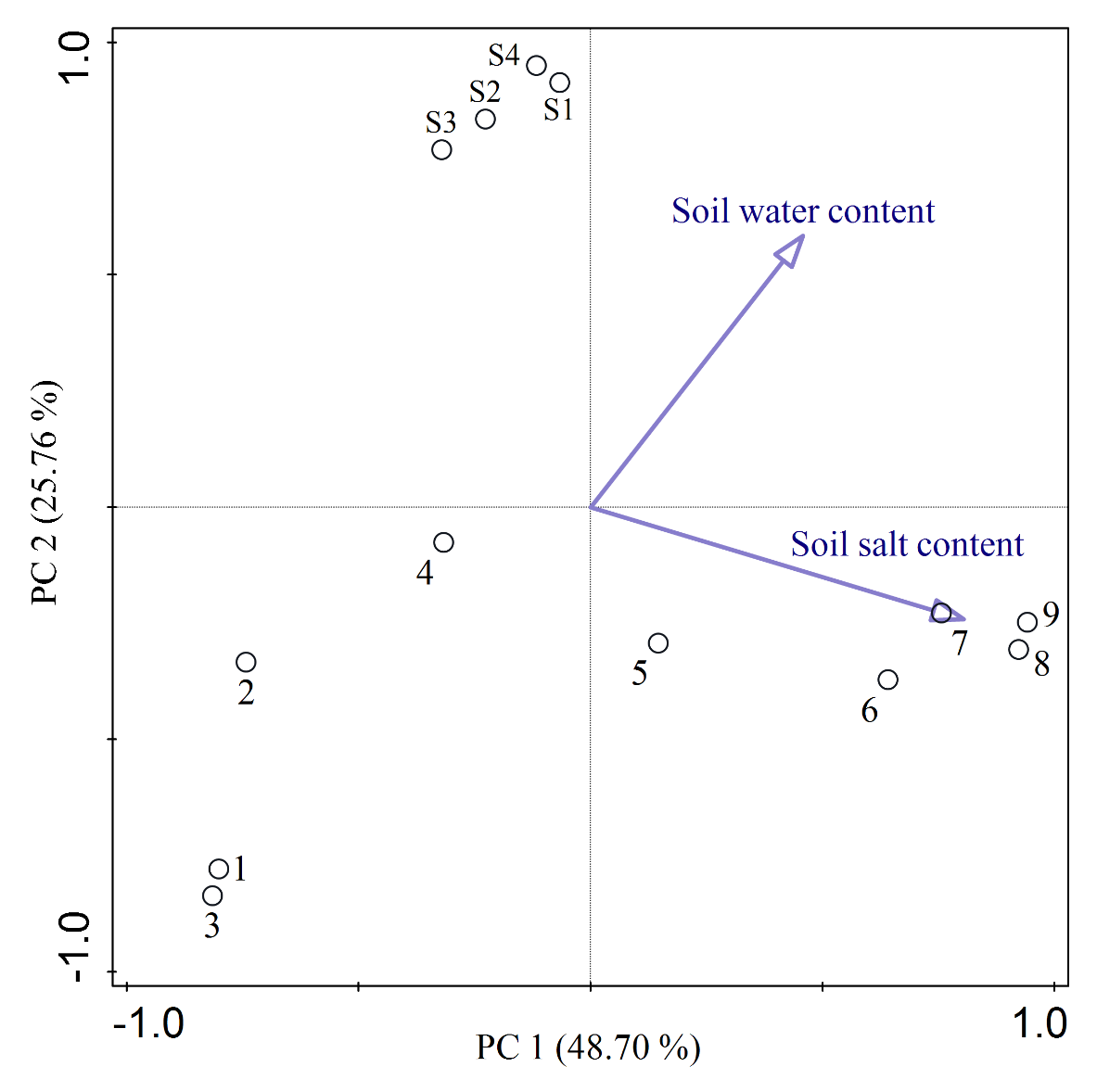


**Figure S2** Principal component analysis (PCA) for the 13 sites by the soil data. Sites 1, 2, 3 and 4, sites 5, 6, 7, 8, and 9, and sites S1, S2, S3 and S4 were defined as dry sites, humid-saline sites and humid-non-saline sites, respectively.





**Figure S3** Comparisons of soil water, salt, organic carbon, total N and P contents (0–100 cm) between dry, humid-saline sites and humid-non-saline sites, respectively (error bars denote SE). Following ANOVA, multiple comparisons were conducted using LSD; any two samples with a common letter (a, b or c) are not significantly different (P>0.05).





**Figure S4** Influence of aridity and salinity on leaf N, P and N:P ratio across different site types (error bars denote SE). Significant differences among dry sites, humid-saline sites and humid-non-saline sites were identified from ANOVA. Following ANOVA, multiple comparisons were conducted using LSD; any two samples with a common letter (a, b or c) are not significantly different (P>0.05).
